# Satellite telemetry and social modeling offer new insights into the origin of primate multilevel societies

**DOI:** 10.1038/ncomms6296

**Published:** 2014-10-22

**Authors:** Xiao-Guang Qi, Paul A. Garber, Weihong Ji, Zhi-Pang Huang, Kang Huang, Peng Zhang, Song-Tao Guo, Xiao-Wei Wang, Gang He, Pei Zhang, Bao-Guo Li

**Affiliations:** 1 College of Life Sciences, Northwest University, Xi’an 710069, China; 2 Anthropology Department, University of Illinois, Urbana, Illinois 61801, USA; 3 Institute of Natural Resource, Massey University, Albany 0632, New Zealand; 4 School of Sociology and Anthropology, Sun Yat-Sen University, Guangzhou 510275, China; 5 Institute of Zoology, Shaanxi Academy of Sciences, Xi'an 710032, China

## Abstract

Multilevel societies (MLS), in which polygynous reproductive units are nested in a larger social matrix, represent a highly complex social system documented only in a small number of mammalian species. Using long-term behavioural data, satellite telemetry and social network analysis, we present a new framework for understanding the function and social dynamics of the golden snub-nosed monkey MLS. Here we show that several one-male units form a cohesive breeding band that associates with one or more all-male units to form a herd. Herds seasonally aggregate and exchange members, thus facilitating gene flow and inbreeding avoidance. This MLS evolved from the aggregation of independent one-male, multifemale units that characterize ancestral Asian colobines; the evolutionary pathway leading to this MLS contrasts with that proposed for African papionins, which appear to have undergone internal fissioning of multimale–multifemale groups. The results suggest that both environmental and phylogenetic factors are important in the evolution of a primate MLS.

## Abstract (web summary)

Multilevel societies (MLS) are a complex form of group organization found in mammals, yet the origin of MLS is poorly understood. Here the authors show that MLS of the golden snub-nosed monkey evolved from the aggregation of independent one-male, multifemale units found in ancestral Asian colobines.

## Graphical abstract

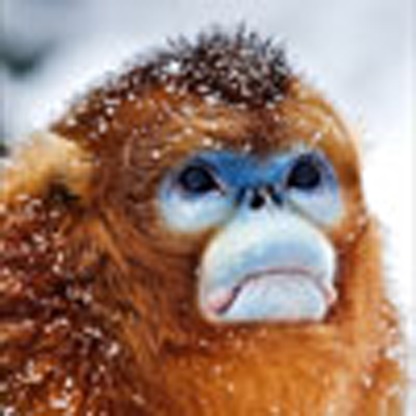

The diversity of animal social systems represents an adaptive response to environmental and social pressures experienced during their evolutionary history^1^,^2^,^3^. Multilevel or modular societies (MLS) represent a complex form of group organization composed of several independent social units nested within a larger social matrix of several hundred individuals^4^. Although rare among mammals, MLS are found in elephants, equids, whales and a small number of primate species^5^,^6^,^7^,^8^,^9^. Primates are noteworthy in exhibiting enhanced cognitive abilities, which appear to have evolved in response challenges associated with living in a complex and changing social environment^10^,^11^,^12^. Most primate species live in social groups containing 4–30 individuals that occupy partially exclusive home ranges or are territorial and generally avoid each other^2^,^13^. However, primate MLS differ from this pattern in that they are comprised of several socially and spatially distinct one-male, multifemale units (OMUs) that coordinate their activities and feed, forage, rest and travel together to form a single large band. This social system has only been reported in geladas (*Theropithecus gelada*), hamadryas baboons (*Papio hamadryas*), some Asian colobines (in particular snub-nosed monkeys of the genus *Rhinopithecus* spp.) and humans^14^,^15^,^16^.

Despite certain similarities in the structural properties of primate MLS, studies conducted over several decades on geladas and hamadryas baboons revealed that the underlying social dynamics of their MLS are fundamentally different^9^,^17^. In geladas, several OMUs aggregate to form a band, characterized by male dispersal and female philopatry within their natal OMU^18^. Strong female kin-bonds within a matrilineal harem play an important role in social cohesion^19^. Bands may merge to form a large community of up to 600 individuals^20^. In the case of hamadryas baboons, however, two or three OMUs spatially associate to form a patrilineal clan^21^,^22^. Males commonly remain within their natal clans and females transfer between OMUs and clans^23^. Several clans aggregate to form a band, OMUs rarely transfer between clans or bands^22^. In contrast to geladas, female hamadryas baboons develop stronger social relationships with their residential male than with other harem females^24^.

Although there is no consensus as to the set of factors that selected for primate MLS, two evolutionary pathways for MLS have been proposed^25^,^26^. In gelada and hamadryas baboon, ancestral mixed-sex multimale multifemale groups of African papionins appear to have undergone a process of internal fissioning resulting in the formation of harem-based mating units nested within the band (Fission Model). Enhanced sexual dimorphism in body mass, hair patterns and pelage colour in African papionins resulting from intrasexual competition may have enabled individual males to improve their breeding success by maintaining exclusive access to several female mating partners^27^,^28^,^29^,^30^. This Fission model assumes that a change in the spatial dispersion of feeding sites into small and scattered patches coupled with male monopolization of females^26^,^31^ are the primary drivers of a MLS.

More recently, evidence of the existence of a MLS in Asian colobines of the genus *Rhinopithecus* has offered new insights into the origin and evolutionary history of modular societies. In snub-nosed monkeys, band size ranges from 50 to several hundred individuals distributed into four to >25 OMUs, one or more all-male units (AMUs) composed of sub-adult and adult males and solitary males^32^. This is in marked contrast to the size and social organization found in other Asian colobines, in which single OMUs of 7–20 individuals living in separate home ranges are common^33^. Researchers have proposed that social pressures associated with reproductive competition by invading bachelor males^34^ and ecological pressures associated with efficiently locating and harvesting widely scattered and seasonally limited resources^35^ have selected for the semi-permanent aggregation of several OMUs into a large MLS (Fusion Model).

Snub-nosed monkeys (genus *Rhinopithecus* spp.) include five species of endangered leaf monkeys that inhabit the northern-most distribution of Asian colobines. These primates exploit seasonal mountainous temperate and subtropical forests at an altitude of up to 4,500 m in China, Vietnam and Myanmar^36^,^37^. Although early accounts of snub-nosed monkeys suggested that they lived in aggregation of extremely large number of social units^38^, difficulties in habituating and following identified individuals across their large and rugged mountainous terrain made direct observations almost impossible^39^, leading to a limited understanding of the dynamics of their MLS.

Long-term observations of golden snub-nosed monkeys (*Rhinopithecus roxellana*) at one site in the Qinling Mountains of north central China, have resulted in detailed data on the social organization of a breeding band. Initial research revealed that each OMU represents a socially close-knit and cohesive unit that engages in few direct social interactions with other OMUs that are embedded in the same band^40^,^41^. Studies of affiliation patterns between individuals within OMUs showed that both intersexual bonds and female-female kin-bonds contribute to the maintenance and cohesion of an OMU^42^,^43^. There also is evidence that adult and sub-adult females transfer between OMUs of the same band, whereas males tend to disperse between bands^41^,^44^,^45^. In this regard patterns of individual transfer and social affiliation in golden snub-nosed monkeys differ from that reported in the matrilineal social units of geladas or the patrilineal clans of hamadryas baboons^44^,^46^.

Given the fact that snub-nosed monkey breeding bands utilize extremely large home ranges (20 km^2^) and that neighbouring bands rarely come into contact^47^, a critical set of questions in understanding the evolution and dynamics of the snub-nosed monkey MLS concerns how do neighbouring bands partition the landscape, whether inter-band relations are best characterized by attraction or avoidance, and how do AMUs and solitary males move in relation to each other and neighbouring breeding bands. In previous years, we have noted several cases in which individuals from outside the band have migrated into the study band. This includes individual adult males who replaced the residential male, fully formed OMUs that entered the band and unidentified females who entered the band and joined existing OMUs (Fig. 4). The origin of these immigrants was unknown. Beginning in 2003, we encountered a neighbouring breeding band (DJF) as well as their satellite AMUs that occasionally merged with our study band into a single large troop of over 300 individuals. Limited evidence indicated tolerance between the two bands when they merged.

Over the course of several years, we observed neighbouring bands at our site to occasionally fuse and we recorded unknown individuals entering our primary study band. This led us to hypothesize that in golden snub-nosed monkeys two or more breeding bands, their satellite AMUs and solitary males temporally aggregate to form the West Ridge Troop (WRT). The function of this troop-level social grouping appears to facilitate the exchange or transfer of individuals between otherwise isolated breeding bands. We refer to this as the ‘Gene Flow Hypothesis’. Due to logistical constraints associated with following multiple bands of golden snub-nosed monkeys simultaneously across steep mountainous terrain, we fitted global positioning system (GPS) collars on five adult males, each from a different component of the snub-nosed monkey MLS: (1) the GNG-breeding band, composed of 13 OMUs with 138 individuals of both sexes, (2) the GNG-all-male band, composed of 3 AMUs with 28 bachelor males, (3) the DJF-breeding band, composed of 7 OMUs with 87 individuals of both sexes, (4) the DJF-AMU, composed of 16 bachelor males and (5) a solitary adult male that followed the GNG-breeding band. We integrated 14 years of monitoring the social dynamics of golden snub-nosed monkeys in the Qinling Mountains, Zhouzhi, China with satellite telemetry and social network analysis (SNA) to record the spatial associations and movement patterns of these five social components, to identify the underlying structure and function of the multilevel society of the golden snub-nosed monkey.

The results demonstrate that the golden snub-nosed monkey MLS is organized into a nested series of expanding social relationships including the OMU, AMU, band, herd and troop. Each level is characterized by different patterns of temporal and spatial association, individual and OMU exchange and differential access to reproductive partners. We hypothesize that the golden snub-nosed monkey MLS evolved from the aggregation of several independent OMUs, into a large and stable band of over 100 individuals that functions to decrease predation risk, promote inbreeding avoidance and enhance gene flow exploiting extremely large home ranges and highly fragmented high altitude mountainous landscapes. We conclude that both phylogenetic and ecological factors play an important role in the origin and evolution of primate MLS.

## Results

### Association pattern among social components of MLS

**Figure 1:**
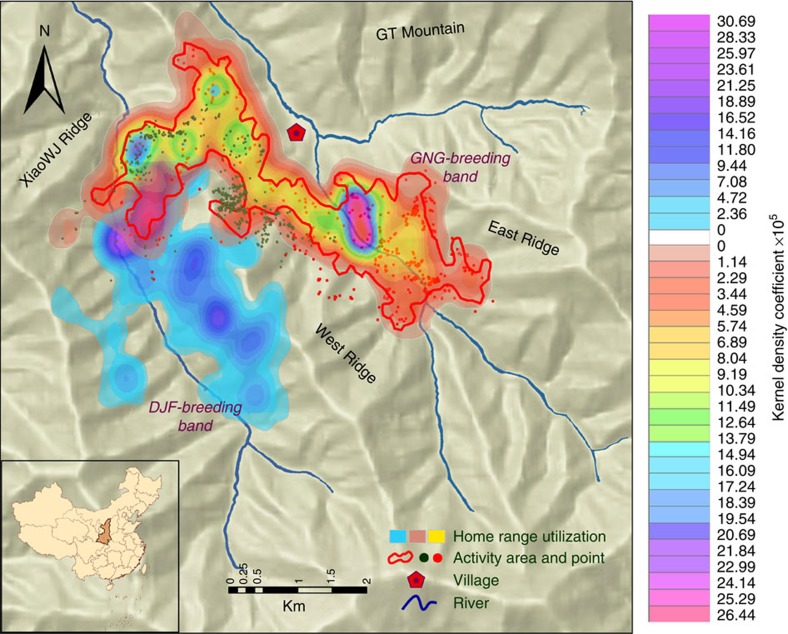
Home range and overlap for each social component of the WRT. The dimensions of the home ranges of the GNG- and DJF-breeding bands are shown by the kernel-based contour lines; the home range of the GNG-breeding band and the DJF-breeding band, was 928.7 ha and 609.1 ha respectively; the area outlined in red represents the boundary of the home range of the GNG-all-male band (762.1 ha), the red octagons represent the home range area (856.7 ha) used by the solitary male in the GNG-herd, and the green teal circles represent the home range area (413.7 ha) occupied by the DJF-AMU.

Based on a Kernel density analysis of location data obtained from the five GPS collars, we found that the home ranges of the GNG-breeding band, the GNG-all-male band and the solitary male overlapped by 72.9% (Fig. 1). The average distance between the GNG-breeding band and the GNG-all-male band was 0.52±0.21 km (0.13–1.53 km), which was significantly smaller than the distance between either social component and the solitary male (One-way analysis of variance: *F*_2, 5109_=240.59, *P*<0.001) (Fig. 1). For example, the distance from the GNG-breeding band to the solitary male was 1.37±0.57 km (0.21–4.41), and the distance from the GNG-all-male band to the solitary male was 1.34±0.58 km (0.03–4.37 km).

**Figure 2:**
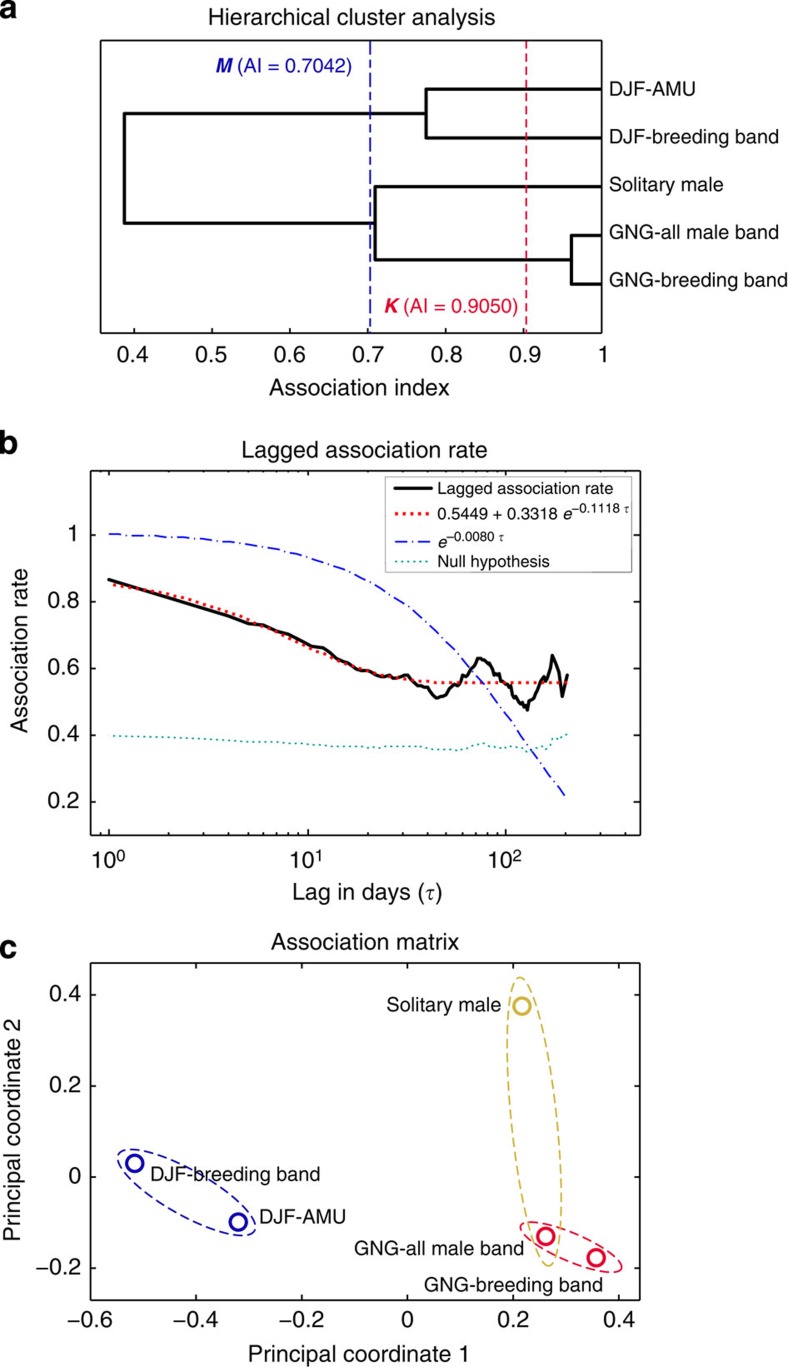
Associations between the social components of the *R. roxellana* WRT at Zhouzhi from 2012 to 2013. (**a**) Association dendrogram; ***M***, the blue-dashed line, is the maximum value showing the modularity of the dendrogram, which represents the recommended cutoff value defining a herd; ***K***, the red-dashed line, based on the knot diagram, provides the cumulative number of bifurcations at different association distances. (**b**) Lagged association rates and null association rates with two fitted models of exponential decay in association. (**c**) Result of the principal coordinates analysis identifying different levels of social hierarchy (based on the association matrix of the half-weighted indexes (HWIs)); distance between social components is inversely proportional to the square root of their HWIs.

In addition, the half-weighted index-based SNA with a 2 h sampling interval confirmed that members of the GNG-breeding band, the GNG-all-male band and the solitary male (Fig. 2) maintained in closer spatial association than each had with the DJF-breeding band and the DJF-AMU. These data indicate that the three GNG-social components consistently coordinated their patterns of movements to form an integrated level of social affiliation, the ‘herd’.

In contrast the DJF-AMU often travelled apart from the DJF-breeding band (mean distance 2.06±1.04 km; range 0.84–4.11 km). Home range overlap between the DJF-breeding band and the DJF-AMU was 12.9% (Fig. 1), and the average distance between the DJF-breeding band and the DJF-AMU was significantly greater than that between the GNG-breeding band and the GNG-all-male band (independent *t*-test, *t*=−53.87; *P*<0.001; degree of freedom (*df*)=3407).

Although the ranging pattern of the DJF-breeding band and DJF-AMU indicates weak social affinity, the demographic evidence shows that most individuals of DJF-AMU were originally members from the DJF-breeding band. SNA also demonstrates that the DJF-AMU and the DJF-breeding band were less closely associated to each other compared with the social components of the GNG-herd (Fig. 2).

### Fission–fusion dynamics of the snub-nosed monkey MLS

**Figure 3:**
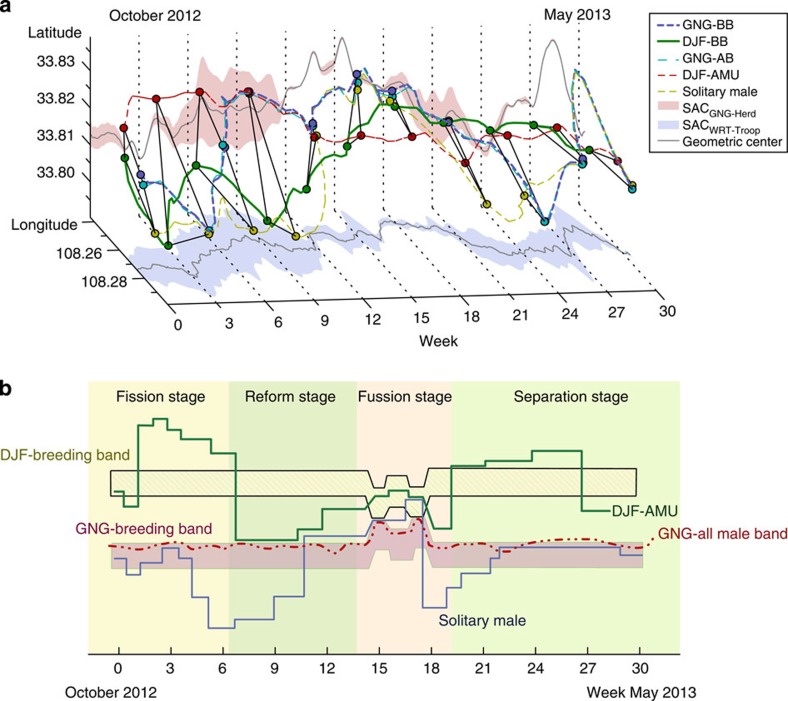
Troop fission–fusion. Using data from the period of September 2012 to May 2013, when all GPS collars were fully functioning, we analyzed spatial/temporal fission–fusion events in the WRT. (**a**). spatial patterns of troop fission–fusion. The lines in this three-dimensional representation are the longitudinal and latitudinal positions of each social component over time. The vertical projection, in red, shows the spatial attraction represented by the spatial affinity coefficient (SAC) values between the GNG-breeding band, GNG-all-male band and solitary male (these comprise the GNG-herd); the horizontal projection illustrates the fission–fusion dynamics and social affinity of all five social components (GNG-herd plus the DFJ-breeding band and DFJ-AMU) in the multilevel society of the WRT-troop. The width of the projection in each time segment represents the SAC value determined by the spatial spread of the social components (**b**). This social dynamics diagram illustrates the four stages categorized using the SAC thresholds. The distance between the lines and stripes represent the degrees of fission–fusion (based on the SAC index) between each social component within the WRT-troop.

Importantly, the telemetry data (Fig. 3a) revealed that during a 3-week period in February and March, the social components represented by the five collared males merged to form a single large troop. Based on the spatial affinity coefficient (SAC) analyses (Fig. 3a,b), we identified four stages of social affiliation that characterized the golden snub-nosed monkey multilevel society: the fission stage, the reform stage, the fusion stage and the separation stage. Our results indicate that these periods accounted for 20.9, 24.7, 18.4 and 36.0% of the study period, respectively) (Fig. 3b). The SACs of the troop during each time segment indicated a lower SAC value during the fusion stage, which means a higher level of troop cohesion, compared with all other stages (Wilcoxon rank sum test, *Z*=4.736, *P*<0.001; *df*=1703).

### Individual exchange between social components of MLS

**Figure 4:**
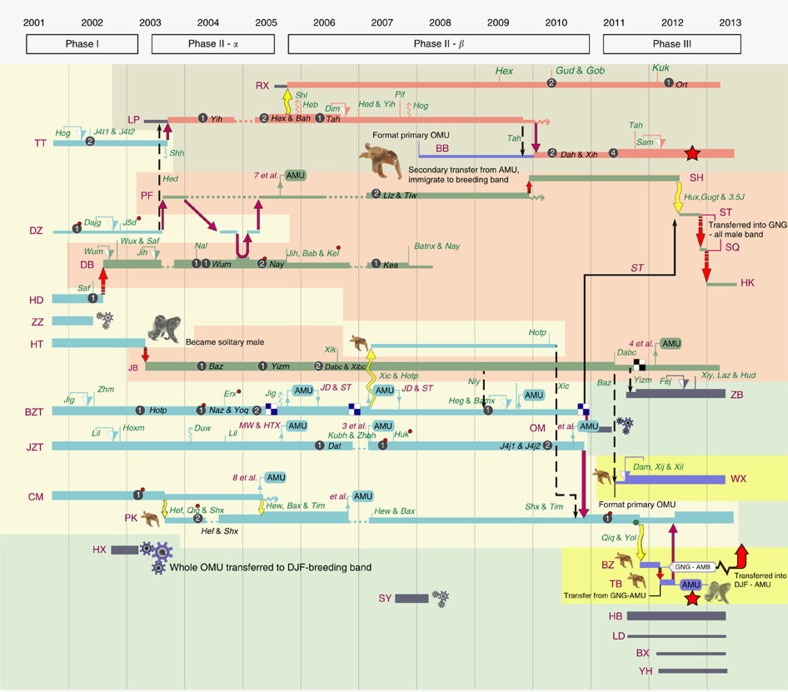
The social dynamics of the GNG-breeding band of the WRT-troop. Each horizontal bar represents the social history of a single OMU in the GNG-breeding from 2001–2013; Capital letters on the left represent the codes of the OMU. The thicknesses of the bars are proportional to the number of adults in the OMU. The light blue stripes are OMUs that existed in the GNG-breeding band when we began observations in 2001. The green bars indicate the OMUs in which the resident male was replaced but otherwise the composition of the harem (we define harem as the set of adult females residing in an OMU) remained the same. The dark grey bars represent OMUs that immigrated into the band. The dark pink bars represent OMUs that increased in size as a result of resident females and their offspring transferring into this OMU; the dark blue bars represent OMUs that were formed when a bachelor male attracted several sub-adult females to form a harem; black-dashed arrows indicate that a single female transferred between harems. Yellow arrows indicate that several members of a harem transferred into a new harem. Purple arrows signify that an entire harem transferred to a new leader male. Red arrows represent those cases in which an entire harem was taken over by a new leader male. Solid-line Black arrows represent male transfer within the band.

Although the telemetry data do not provide details of individual movements, we have accumulated evidence of individual dispersal patterns between the social components of the WRT based on 14 years of field observations. During this period, we identified 127 individuals (73 emigration events and 54 immigration events) and 20 OMU transfers that occurred between the GNG-breeding band and the DJF-breeding band. In 14 of 20 cases in which an entire OMU from the DJF-breeding band immigrated into the GNG-breeding band, it remained in the band for a period of at least 3 months (Fig. 4). We also identified nine bachelor adult males from the GNG-all-male band and the DJF-AMU who successfully immigrated into the GNG-breeding band on their own, and formed an OMU, either by attracting females or by taking over an existing OMU. We recorded 14 resident males who lost their tenure as the leader male of an OMU in the GNG-breeding band. Seven of these cases involved a takeover by an immigrant male, and in these instances, the ex-harem leader joined the GNG-all-male band on four occasions, one male transferred into the DJF-AMU, one became a solitary male and one died soon after he was replaced. We also recorded a male from the GNG-all-male band becoming a harem leader in the GNG-breeding band, losing his harem after 4 months and then transferring back into the GNG-all-male band. This male disappeared from the GNG-all-male band and 6 months later was observed as a member of the DJF-AMU (Fig. 4).

## Discussion

**Figure 5:**
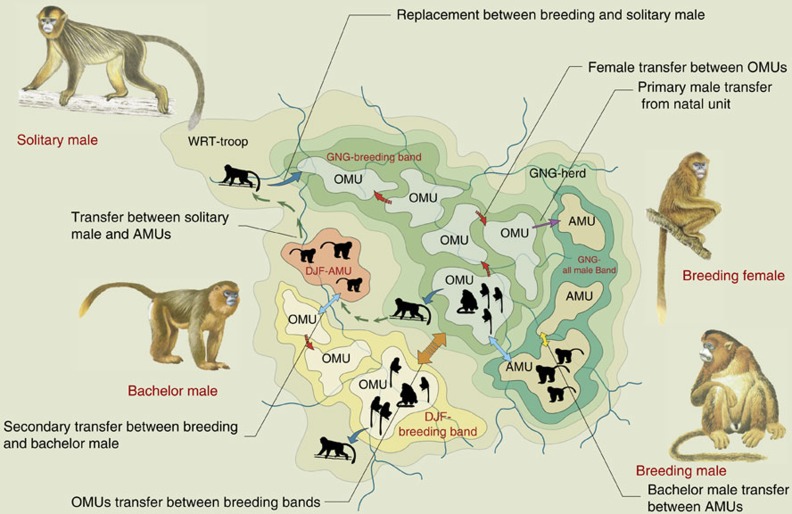
The multilevel society of the Golden snub-nosed monkey. The fundamental level of social organization is the one-male, multifemale social unit (OMU), which contains a single breeding male, several breeding females (harem) and their offspring. There also exist all-male units (AMU), in which several bachelor males reside together. Many of these are juvenile and sub-adult males who transferred directly into the AMU from their natal OMU. In some cases adult AMU members are previous residential males of an OMU. AMU members are characterized by an age-graded dominance hierarchy and kin relationships. Solitary males are principally adults who have been replaced as breeding males in their OMU. However they may transfer into an all-male band or follow the breeding band and try to take over an OMU from the residential male. The breeding band is an aggregation of OMUs that coordinate their daily activity. Although they feed, forage, rest and travel together in a coordinated manner, members of different OMUs within the breeding band rarely engage in social interactions. The all-male band is composed of 1–3 AMUs that are socially distinct from each other but coordinate their activities in close spatial proximity. The herd is composed of an associated breeding band, all-male band and solitary males. Although the social components within a herd are often separated, they are characterized by considerable home range overlap and coordinate travel. The troop is composed of two or more herds in a large home range that engage in seasonal fission–fusion dynamics. Individuals and OMUs have been observed to transfer between herds of this multilevel society. The snub-nosed monkey illustrations are copyrighted 2014 by Stephen D. Nash/IUCN/SSC Primate Specialist Group. Used with permission.

These data reveal, for the first time, the complex social dynamics of the golden snub-nosed monkey multilevel society (Fig. 5) and confirm the process of the fusion of two herds to form a troop. During troop fusion, members of different breeding bands have the opportunity to exchange individuals across social components that were previously regarded as socially and reproductively separate or distinct. Thus, the multilevel society of the golden snub-nosed monkeys is best described as a system of nested social, spatial and reproductive relationships among individuals forming distinct OMUs that merge into a cohesive breeding band, an associated or satellite all-male band (or AMUs) and solitary males. The breeding band and associate satellite AMUs and solitary male form a herd-level of social and spatial organization. Neighbouring herds periodically fuse to form a troop. The formation of this highly inclusive multilevel social organization appears to facilitate gene exchange among otherwise relatively isolated golden snub-nosed monkey populations.

The results of this study offer new insights into the evolution and maintenance of this primate MLS and serve to differentiate the set of social and ecological factors that selected for the formation of the *Rhinopithecus* breeding band and troop. Phylogenetic reconstructions based on mitochondrial DNA data and paleobiogeography indicate that *Rhinopithecus* diverged from the other genera of odd-nosed monkeys during the late Miocene, and then differentiated into five species^48^. This appears to have begun ~2 million years ago in response to oscillations in paleoclimate associated with the uplifting of the Tibetan Plateau, a decrease in temperature and rainfall, a reduction in broadleaf and evergreen forest habitats, glacial expansion and geographical barriers that restricted gene flow across populations^49^,^50^,^51^. Although the precise factors that led to the formation of a multilevel society remain unclear, increased environmental heterogeneity and forest fragmentation during periods of glacial maximum may have resulted in feeding sites that were hyper-dispersed across the landscape^52^. This may have favoured the year-round aggregation of separate snub-nosed monkey OMUs, as is found in most Asian colobines, into a larger, more cohesive and socially and spatially complex breeding band^33^,^34^. We term this the fusion model of breeding band formation to distinguish it from the MLS of gelada and hamadryas baboons that is argued to have formed by the internal fissioning of a multimale multifemale social organization into several nested harem reproductive units within a larger band^31^.

Several theories have been proposed to explain the aggregation of individual OMUs to form a band-level social organization in snub-nosed monkeys. These include the bachelor threat hypothesis^34^,^53^, the predator defence hypothesis^54^,^55^ and the inbreeding avoidance hypothesis^43^,^56^.

The bachelor threat hypothesis argues that the formation of a breeding band favoured several harem males acting collectively to form a coalition and defend OMUs against invading bachelor males who threaten to take over a harem^34^. It also had been reported that the bachelor males of geladas monitor the interactions of females and their harem leaders and may use this information to evaluate the strength and quality of harem holders and the cohesiveness of the OMU^57^. In the black-and-white snub-nosed monkey, an AMU was regularly found 200–500 m from the OMUs and occasionally located in the centre of the band within the reach of the reproductive units^32^. Our behavioural observations and satellite telemetry provide the first quantitative and detailed account of the movement patterns of breeding bands, AMUs and solitary males. The GNG-all-male band travelled independently from the GNG-breeding band, but maintained a close spatial association and coordinated their movement patterns, resulting in a high degree of social affinity and spatial overlap. The distance between the all-male band and the breeding band consistently ranged from 100–1,500 m. This distance could be travelled by a bachelor male within the span of several minutes to several hours. Moreover, we observed leader males to form coalitions and aggressively chase members of the all-male band when individuals were in close proximity to the breeding band. This same behavioural pattern has been reported in a study of golden snub-nosed monkeys in Hubei Province, China^58^.

Similar to the threat of bachelor males, some have argued that predation risk posed by large ground predators may have also played a critical role in the evolution of the *Rhinopithecus* breeding band^54^,^55^. If multiple individuals engage in predator detection, this might provide advantages to all members of a cohesive breeding band. Sperm whales (*Physeter macrocephalus*) represent another mammal that lives in a complex nested social organization. Eastern Pacific populations of sperm whales live in small units composed of several unrelated matrilines. These units temporarily merge to form a clan in response to high density of killer whales^59^. In contrast, the same species of sperm whales in the North Atlantic rarely group with other social units and do not form clans due to reduced predator pressure. The results argue that protection against predation by killer whales in the eastern Pacific is the primary reason for the formation of a MLS^59^.

Another factor proposed to select for the aggregation of snub-nosed monkey OMUs into a breeding band is inbreeding avoidance. It had been reported that under conditions in which the breeding tenure of a harem male exceeds 4–5 years, female golden snub-nosed monkeys transfer from their natal OMU into another OMU in the breeding band to avoid mating with their father^43^. An advantage of nested OMUs within a breeding band is that it reduces the cost of female transfer by allowing sub-adult and young adult females to move directly from one OMU in the breeding band to another^43^. Finally, food distribution may also have contributed to the evolution of large multilevel groupings^34^. In the temperate forests of snub-nosed monkey habitat, evenly distributed food resources allow a larger number of OMUs remain in close proximity within a modular society^34^,^40^. In contrast, most Asian colobines that live in habitats characterized by patchy food distribution form small territorial social units, which has shown agonistic inter-unit interactions and food defence^33^,^60^,^61^.

We also provide quantitative evidence for a new level of social organization within the MLS of golden snub-nosed monkeys, the troop. Based on spatial analysis of GPS monitoring data, we documented the temporary fusion of two herds during a limited period of the year (February and March). The fact that both individuals and entire OMUs transfer between herds provides an opportunity for gene flow across social components living under conditions of low population density (<10 individual per km^2^) and distributed across large home ranges in mountainous habitats^33^. However this troop-level social organization is not stable, likely in response to increased feeding and reproductive competition among the 20 OMUs and the 44 bachelor males that compose the troop. In our study the fusion stage of troop formation coincided with a period of local food abundance and the flushing of buds and young leaves, which are high in protein^47^,^62^. The formation of a large troop has also been reported in the Grey snub-nosed monkey (*Rhinopithecus brelichi*), which are reported to contain as many as 600 individuals who aggregate in spring/early summer but break down into smaller groups during most of the year^63^.

In summary, our results using satellite telemetry, SNA and long-term behavioural observations demonstrate that snub-nosed monkeys live in a modular society organized into several levels of association. In this system the basic elements are solitary males, OMUs and AMUs. Breeding bands consist of an aggregation of several OMUs and all-male bands consist of an aggregation of several AMUs. These bands represent socially and spatially distinct components of the MLS. A herd is defined as the spatial aggregation of the breeding band, an all-male band (or single AMU), and solitary males. Previous studies have used the term band to describe the social and spatial association of several OMUs and AMU^26^,^36^. Using long-term observations and satellite telemetry, we found that an all-male band or single AMU, as well as solitary males travel independently from the breeding band during most part of the year. Therefore, we use the term herd to document the association between different breeding bands and all-male bands. The classification we use to distinguish the band-level and the herd-level is based on analysis of SNA (Fig. 2a), which indicated a statistical threshold to highlight patterns of social and spatial affinity among these different social elements. During a brief period of the year, two neighbouring herds fuse to form a troop in which individuals and OMUs transfer between otherwise isolated reproductive units.

We documented that each structural component of the snub-nosed monkey MLS appears to serve a distinct function: OMUs represent the main reproductive units; band members experience mutual benefits from leader males collectively acting against challenges from bachelor males, a reduction in predation risk and inbreeding avoidance by the ability of females to transfer directly between OMUs within the breeding band; and the fusion of two herds to form a troop enables individuals from different breeding bands to exchange membership and promotes gene flow between otherwise isolated breeding bands that travel across extremely large home ranges and inhabit highly fragmented and heterogeneous high altitude temperate forest habitats.

In line with Grueter and van Schaik^34^, we suggest the MLS of *Rhinopithecus* evolved by the aggregation of separate OMUs from an ancestral Asian colobine into a larger, more cohesive breeding band, which is an alternative evolution pathway compared with the MLS of African papionins from the internal fissioning of a multimale multifemale social organization. We conclude that both phylogenetic and ecological factors have played an important role in the origin and evolution of a primate MLS. Additional field-based observational data on individual interactions among different tiers of the MLS are needed to augment our understanding of the MLS of *R. roxellana*.

## Methods

### Study subject and social dynamics

Since 1999, we have been investigating the social structure of WRT of Golden snub-nosed monkeys in the Zhouzhi National Reserve in central China^43^. The WRT is composed of the GNG-breeding band, the GNG-all-male band, the DJF-breeding band, the DJF-AMU and several solitary males. We habituated the GNG-breeding band in 2001 using a method of semi-provisioning^44^. The home range of the GNG-breeding band encompasses 2250, ha of mountainous forest, ranging from deciduous broadleaf to coniferous forest (1,380–2,974 m; ref. 47). Each individual was fitted with a RFID identification tag (TX1411SSL) subcutaneously in the arm, and tattooed with an individually unique sequence of numbers and colours (red, blue) on the upper or lower area of its lips. This enabled us to unambiguously identify each individual and monitor both short-term and long-term dispersal events. We also used micro-satellite DNA markers to confirm the kinship and individual identification.

### GPS location and collars configuration

To more accurately monitor band movements, the location and duration of troop fusion and fission dynamics, and patterns of female, male and OMU inter-band transfer in August 2012 we fitted five fully adult males with GPS collars (7000SLU series, LOTEK Wireless Inc., Canada). Based on observations conducted over several years, each male was selected to represent one of the independent social components of the WRT.

GPS Collars were configured to automatically locate every 2 h from 0500 to 1900, h for a period of 104 weeks. After that time, the collars were programmed to unlock and release from the animal’s neck using the Time Drop-off feature of the collar.

The collars recorded the location (in universal transverse mercator coordinates) and elevation of the target golden snub-nosed using a system of seven satellites. The data were stored in the collar’s memory chip. We used a BIOTRACKER receiver (LOTEK Wireless Inc.) to track each individual male by VHF beacon signals. Once we were in the vicinity of the band, we used a hand-control wireless unit (LOTEK Wireless Inc.) to download previous location data via a UHF radio signal. Location data from each GPS collar were collected during 291 days (September 2012 to May 2013). Within this study period, each of the social components of the MLS travelled freely across their home range without any interference by the researchers.

### Range pattern data analysis

We used the dilution of precision algorithm to determine the level of accuracy for each location data point. We eliminated location data for which dilution of precision≥8 (2.0% of all). In an additional 2.7% of the records, prevailing weather conditions or other factors resulted in the collars failing to record the location for a given time interval. In these cases we used the piecewise cubic spline Interpolation method to reconstruct the data for the empty time segment.

Location data obtained were analyzed using the Kernel density analysis of home range utilization and spatial overlap estimation tools from ArcGIS 10.0 (Esri, USA) to determine the ranging patterns of the golden snub-nosed monkey.

### Fission–fusion dynamics and satellite telemetry modelling

To reduce the random error of GPS locations, we calibrated the GPS coordinate data using a smoothing function for each of the five individuals, representing a different social component of the snub-nosed monkey MLS,


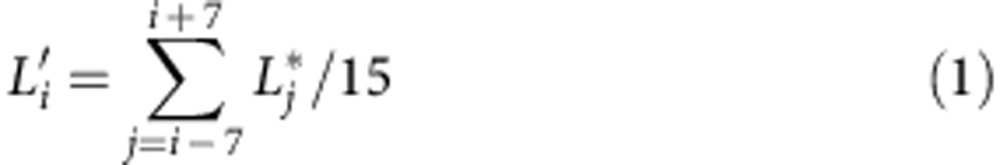


Where 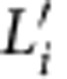 is the smoothed location calibrated from an average of 15 GPS points per collar; 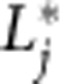 is the uncorrected location data downloaded from the GPS collars, with *i* as the time variable and *j* as the loop variable.

To estimate the spatial dispersion pattern and fission–fusion dynamics of the five social components, we used the SAC as a measure of social attraction.

With,


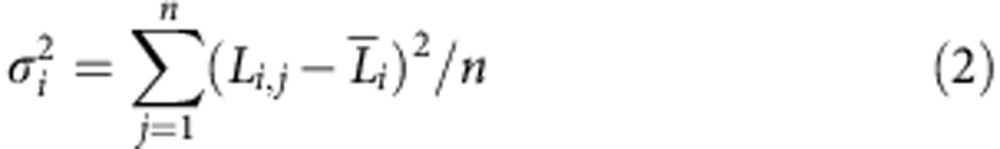


the value *σ*^2^ represents the variance of the GPS coordinates among the *n* social components in one dimension (longitude or latitude) within the same time segment. *L*_*i,j*_ denotes the *j*th spatial coordinate in one dimension at time=*i*, and 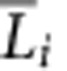 is the mean value of *n* spatial coordinate points at time=*i*.

By taking the modular of the s.d. vector 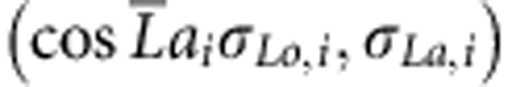



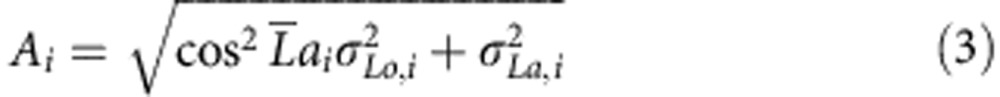


we are able to measure social attraction in space of *n* social components at a given time segment. Then we standardize the *A*_*i*_ by


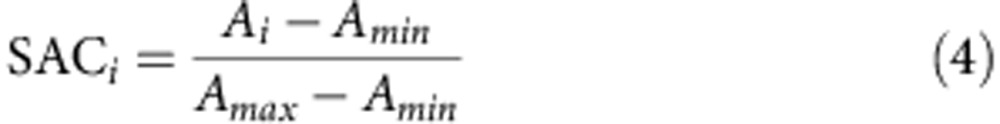


Where SAC_*i*_ represents the standardized *A*_*i*_ and ranges from 0 to 1, with SAC approaching zero, indicating a tendency for greater spatial fusion, and SAC approaching 1, indicating a tendency for greater spatial fissioning. The SAC values are also presented as the width of the vertical and horizontal projections of each time segment in Fig. 3a.

To measure quantitatively fission and fusion events across the study period, we calculated the threshold of dispersion based on the SAC values. We assume that the golden snub-nosed monkeys could continue to effectively communicate within a maximum spatial range of 513.12 m. This distance was determined based on a calculation of the average maximum distance recorded between OMUs within the GNG-breeding band (1,726 h of data collected using instantaneous scan sampling that recorded the distance between each OMU dyad within the GNG-breeding band from 2003–2007). We conservatively used the maximum distance between any two OMUs (OMU dyad) for each scan to calculate the average distance. We also assumed that the spatial pattern of all social components within a multilevel society at time segment=*i* fits the shape of a geometric polygon with each social component represented as a node. Therefore, the fission and fusion threshold values of SAC were calculated based on the assumption that the length of each side of the polygon is equal to the distance of effective communication.

With


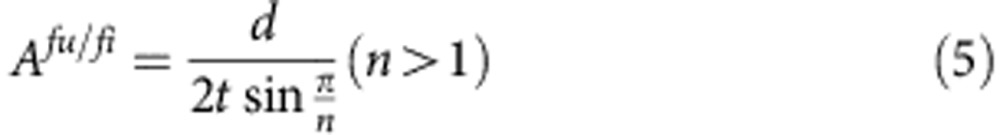


Where *d* is the length of the sides of an isogon (equilateral polygon) with *n* sides (here *n*=5 as there were five social components within the studied troop). The *A*^fu/fi^ is the radius of the circumcircle that fits the isogon. This value is equal to the s.d. of the spatial coordinate value of *n* nodes in two dimensions. Where *t*=the radius of earth (*r*) multiplied by 2*π*/360°. By substituting *A*^fu/fi^ into equation (4), thefission or fusion thresholds of SAC were calculated.

The threshold of SAC^fission^=0.1139 was based on the calculation of an isogon with sides of 513.12 m (distance of effective communication). We defined the fusion stage as occurring when the SAC_*i*_ values for all five social components≤SAC^fusion^ and this spatial association continued for more than three time segments (≥4 h). In contrast, the threshold of SAC^fission^=0.4571 was identified when the sides of the isogon were equal to twice the distance of effective communication (2 × 513.12 m, which we assume is beyond visual and vocal communication). We defined the fission stage when the SAC_*i*_ was greater than the SAC^fission^ threshold and continued for more than three time segments (≥4 h). The reform stage occurs after a fission stage when SAC^fusion^≤SAC_*i*_≤SAC^fission^ and before the next fusion stage. Finally, the separation stage was defined as SAC^fusion^≤SAC≤SAC^fission^ and followed the fusion stage and continued until the next fission stage.

Calculation of the smoothing function, distance function and SACs were performed using Matlab 7.0 (MathWorks, USA).

### Multilevel association estimate and SNA

To better understand the dynamics of troop formation, stability and their social relationships among different social components, we used SNA to measure affiliative relationships based on patterns of spatial/temporal association (Fig. 2).

Affiliation using SNA is divided into association and interaction, where association is defined in terms of spatial proximity^65^,^66^. For this analysis, we calculated the distances between each dyadic social components to identify the nearest neighbour in time segments of 2-h intervals using the distance function,


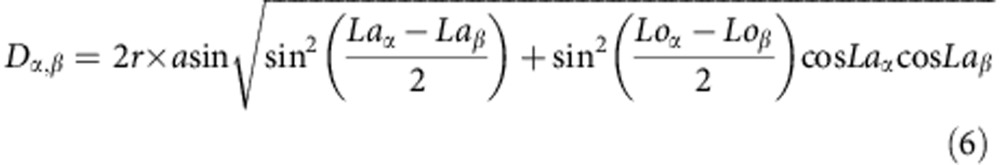


*D*_*α,β*_ is the distance between locations *α* and *β,* La and Lo denote the latitude and longitude of the spatial coordinate of each location, and the radius of earth (*r*) was set to 6378.137 km in all calculations. The sampling period was October 2012 to May 2013.

We used the half-weighted Index (HWI)^67^,^68^ to measure the association between each individual OMU member using SNA.


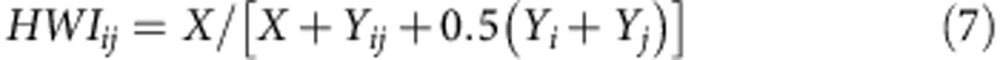


Where *X* is the number of the observation samplings in which social component *i* and *j* were each other’s nearest neighbour; *Y*_*i*_ or *Y*_*j*_ is the number of observations that social component *i* or *j* was the nearest neighbour of social component *j* or *i*, but social component *j* or *i* was not the nearest neighbour of social component *i* or *j*; *Y*_*ij*_ is the number of simultaneous observations that social components *i* and *j* had other nearest neighbours.

Hierarchical cluster analysis^65^,^66^ presented by the dendrogram shows the degree of associations between each social component. The cutoff value^6^,^68^,^69^ in the dendrogram provide quantitative threshold to identify the herd-level social grouping in the study troop. To estimate the stability of troop cohesion of the two estimated herd during sampling periods, we used temporal analysis^67^ presented by lagged association rate to predict the probability of decay in the association rate; null association rate proposed value of the lagged association rate if there is no preferred association. We also used principal coordinate analysis to identify hierarchies of different social components coordinated with each other. All statistical and graphical analyses of SNA were performed using SOCPROG 2.1 (ref. 6).

### Standardized statistic analyses

Standardized statistic analyses were performed in SPSS 16.0 (SPSS Inc., USA). In all analyses, significance level was set at *α*=0.05, average values are expressed as mean±s.e. Sampling in each test were independent.

### Research protocols

All research protocols reported here adhered to the regulatory requirements of and approved by the animal care committee of the Wildlife Protection Society of China (SL-2012-42). The comfort and security of collars devices received clearance from and complied with the protocols approved by the specialist committee of the State Forestry Administration of China (SFA: LHXZ-2012-2788), Chinese Academy of Science and China Duty.

## Author contributions

X.-G.Q. and B.-G.L. conceived and designed the research; X.-G.Q. and Z.-P.H. performed the fieldwork; X.-G.Q., K.H. and Z.-P.H. analyzed the data; all authors contributed to the writing and editing of this manuscript.

## Additional information

**How to cite this article:** Qi, X. G. *et al.* Satellite telemetry and social modelling offer new insights into the origin of primate multilevel societies. *Nat. Commun.* 5:5296 doi: 10.1038/ncomms6296 (2014).
